# Multimodal neuroimaging of frontal white matter microstructure in early phase schizophrenia: the impact of early adolescent cannabis use

**DOI:** 10.1186/1471-244X-13-264

**Published:** 2013-10-17

**Authors:** Denise Bernier, Jacob Cookey, David McAllindon, Robert Bartha, Christopher C Hanstock, Aaron J Newman, Sherry H Stewart, Philip G Tibbo

**Affiliations:** 1 Department of Psychiatry, Dalhousie University, 5909 Veterans’ Memorial Lane, Abbie J. Lane Building, Room 3030, Halifax B3H 2E2, Nova Scotia, Canada; 2 Robarts Research Institute, Western University, 100 Perth Drive, London N6A 5K8, Ontario, Canada; 3 Department of Biomedical Engineering, University of Alberta, 8308-114 Street, Edmonton T6G 2V2, Alberta, Canada; 4 Department of Psychology and Neuroscience, Dalhousie University, Box 15000, Life Sciences Centre, B3H 4R2 Halifax, Nova Scotia, Canada

**Keywords:** Schizophrenia, Cannabis, White matter, *N*-acetylaspartate, Oligodendrocytes, Transverse relaxation time constants, Proton magnetic resonance spectroscopy, Diffusion tensor imaging

## Abstract

**Background:**

A disturbance in connectivity between different brain regions, rather than abnormalities within the separate regions themselves, could be responsible for the clinical symptoms and cognitive dysfunctions observed in schizophrenia. White matter, which comprises axons and their myelin sheaths, provides the physical foundation for functional connectivity in the brain. Myelin sheaths are located around the axons and provide insulation through the lipid membranes of oligodendrocytes. Empirical data suggests oligodendroglial dysfunction in schizophrenia, based on findings of abnormal myelin maintenance and repair in regions of deep white matter. The aim of this *in vivo* neuroimaging project is to assess the impact of early adolescent onset of regular cannabis use on brain white matter tissue integrity, and to differentiate this impact from the white matter abnormalities associated with schizophrenia. The ultimate goal is to determine the liability of early adolescent use of cannabis on brain white matter, in a vulnerable brain.

**Methods/Design:**

Young adults with schizophrenia at the early stage of the illness (less than 5 years since diagnosis) will be the focus of this project. Four magnetic resonance imaging measurements will be used to assess different cellular aspects of white matter: a) diffusion tensor imaging, b) localized proton magnetic resonance spectroscopy with a focus on the neurochemical *N*-acetylaspartate, c) the transverse relaxation time constants of regional tissue water, d) and of *N*-acetylaspartate. These four neuroimaging indices will be assessed within the same brain region of interest, that is, a large white matter fibre bundle located in the frontal region, the left superior longitudinal fasciculus.

**Discussion:**

We will expand our knowledge regarding current theoretical models of schizophrenia with a more comprehensive multimodal neuroimaging approach to studying the underlying cellular abnormalities of white matter, while taking into consideration the important confounding variable of early adolescent onset of regular cannabis use.

## Background

### Rationale

One neurodevelopmental model of schizophrenia [1] postulates a ‘two-hit’ hypothesis. A ‘first hit’ is said to disrupt the trajectory of normal neural development, rendering the brain vulnerable to a ‘second hit’ which then precipitates the onset of psychosis. Similarly, another model postulates that an underlying neuropathological vulnerability is necessary but not sufficient for the development of the illness, and that full disease expression may require a trigger such as an environmental or biological stressor [2]. For both models, early age at onset of regular cannabis use may represent one possible ‘second hit’ or biological stressor associated with full disease expression [3-5].

There is a growing body of evidence suggesting that a disturbance in connectivity between different brain regions, rather than abnormalities within the separate regions themselves, are responsible for the clinical symptoms and cognitive dysfunctions observed in schizophrenia [6]. White matter, which comprises axons and their myelin sheaths, provides the physical foundation for functional connectivity in the brain; it is therefore increasingly becoming a focus of research in order to better understand the underpinnings of schizophrenia. Myelin sheaths are located around the axons and provide insulation through the lipid membranes of oligodendrocytes [7]. Several different lines of empirical data [8,9] have suggested oligodendroglial dysfunction in schizophrenia, based on atypical findings in terms of myelin maintenance and repair in deep white matter regions. Observed oligodencrocyte abnormalities in schizophrenia consist of more dispersed arrangement and lower densities [10,11], reduced absolute numbers [10,12,13] as well as aberrant morphology, necrosis and apoptosis along with damaged myelin sheaths [14,15]. This situation is not likely caused by chronic antipsychotic medications as these drugs have been reported to alter the numbers of astrocytes, not of oligodendrocytes [16].

The brain region of interest for this study is the left superior longitudinal fasciculus (SLF), a large bundle of white matter fibre tract located in the frontal lobe, travelling between the dorsal prefrontal and caudal-inferior parietal regions of the brain. Frontal regions undergo substantial myelination during the periods of adolescence and early adulthood [17,18], especially in the bilateral SLF [19]. Abnormal maturation of the SLF in adolescence may thus be crucial in the development of schizophrenia. Several DTI studies have found abnormalities in the SLF in schizophrenia as well as in asymptomatic cannabis users, as reviewed below. We will investigate the SLF microstructure in early phase schizophrenia (less than five years since diagnosis) [20], focusing on the potential role of early adolescent onset of regular cannabis, and using neuroimaging modalities that are sensitive to cellular changes.

### Neuroimaging modalities and review of the literature

#### Diffusion tensor imaging (DTI)

Water diffusion can occur equally in all directions (isotropic diffusion), for example in cerebrospinal fluid where diffusion is not restricted or in brain tissue where water diffusion is restricted similarly in all directions (e.g., gray matter tissue which has a complex cellular structure). Water diffusion is called anisotropic (preferentially diffusing in one direction) where the brain tissue microstructure contains fibres that are aligned (e.g., white matter fibre tracts); in that case, water diffusion will preferentially occur along the axis of the fibre tracts. An ellipsoid model of anisotropic water diffusion tensor (describing linear associations between vectors) can be calculated for each anatomical voxel (the smallest volumetric unit of brain images).

DTI is thus an in vivo brain imaging tool that provides an index of the micro-structural integrity of white matter tissue [21]. Mean diffusivity (MD) provides a general measure of water diffusion without differentiating the direction of diffusivity. Another measure called fractional anisotropy (FA), when found to be high, will indicate a preferred direction of water diffusion in the region of interest [22,23]; when found to be reduced relative to normative data, it broadly suggests reduced white matter integrity [24]. Ultrastructural studies directly comparing DTI parameters with tissue pathology have associated changes in DTI water diffusivity measures with dysmyelination of white matter tracts; other tissue alterations that influence water diffusivity are axonal pathology and changes in cell densities [24].

There have been over 60 studies using DTI to evaluate white matter integrity in schizophrenia [25]. The majority of these studies have focussed on chronic schizophrenia and have reported evidence of multiple areas of white matter disruption most notably in the corpus callosum, prefrontal white matter, SLF, and cingulum bundle [26,27]. There have been fewer DTI studies of early phase schizophrenia. This cohort is however extremely important, as it allows for the investigation of pathology core to the illness, with minimal impact of confounders such as medication, age, and length of time with illness.

##### DTI in early phase schizophrenia

DTI studies of early phase schizophrenia have yielded inconsistent findings. In a review of this literature (2010), it was observed that for each white matter fibre tract that was found abnormal in the clinical sample relative to the normative sample, there was at least one other, negative research report [27]. In more recent studies (2010–2013), the pattern of mixed findings remains, however with fewer reports of negative findings [28,29] than positive findings [30-38]. Altogether, several different white matter tracts have been reported as disrupted in schizophrenia at different stages of the illness, supporting the hypothesis that white matter deficits could possibly be widespread throughout the whole brain [29]. In early phase schizophrenia more specifically, the three white matter tracts most often implicated are the SLF [31,34,37,39-42], the splenium of the corpus callosum [31,33,34,39,40,43-45], and the fronto-occipital fasciculus [25,31,34,37,39,41-43,46].

##### DTI in cannabis users without schizophrenia

Different lines of evidence support the assumption that early cannabis use in a developing (adolescent) brain could be markedly more damaging than in a more mature brain (with ‘early users’ defined as those below age 17 years; [4,47]). In healthy volunteers, a greater detrimental impact of early initiation of regular cannabis use (relative to a later initiation) has been reported for visual reaction times [48], cognitive performance [49], and volumetric brain tissue abnormalities [50].

DTI studies have reported that early adolescent regular cannabis use in otherwise healthy young adults is associated with reduced FA values in white matter tracts involving fronto-temporal connections [51], and with increased mean diffusivity (MD) in the prefrontal section of the corpus callosum [52]. MD quantifies water diffusion in each voxel and is increased when there is reduced white matter integrity. It is thus possible that early onset cannabis use in adolescence might decrease white matter structural integrity in otherwise healthy individuals. The white matter fibre tracts most often reported as abnormal in cannabis users include the SLF [51,53-56], the corpus callosum [52,53,57,58], and more broadly defined temporal [51,53,56,58] and frontal regions [53,54,57].

##### DTI in cannabis users with schizophrenia

A recent review of epidemiological evidence found that onset of cannabis use in early adolescence is associated with a particularly increased risk of developing schizophrenia [47], while the lifetime rate of cannabis use use in adults with schizophrenia is associated with earlier onset of the illness [59].

In early adolescent onset of schizophrenia, cannabis-positive patients showed reduced FA values relative to cannabis-negative patients in several white matter tracts including the SLF [34]. In people with first episode psychosis, cannabis-naive patients had reduced FA in the corpus callosum, relative to patients with early onset of cannabis use and healthy controls [33]. On the other hand, patients with recent onset schizophrenia and early adolescent cannabis use had increased FA values in temporal and frontal regions, relative to healthy controls; no differences were found between controls and patients without early adolescent cannabis use [60].

Although some findings go in opposite direction (decreased and increased FA values), altogether the empirical DTI data supports the assumption of a greater detrimental effects of cannabis on an immature brain in both healthy volunteers and patients with recent onset schizophrenia. In addition to being a potential “second hit” for psychosis in a vulnerable brain, failure to control for this confounding variable could underlie the inconsistent findings in previous DTI studies of early phase schizophrenia and would demand for this variable to be factored into future studies of white matter abnormalities in psychosis.

#### Proton magnetic resonance spectroscopy (^1^H-MRS)

Another neuroimaging technique that will be used in this study is ^1^H-MRS, which will be acquired from the same targeted brain region (the left SLF). The neurochemical of interest is *N*-acetylaspartate (NAA), a free amino acid that produces the most prominent resonance in ^1^H-MRS of the human brain: a peak located at 2.02 ppm on the spectral profile [61].

*In vivo* concentration levels of NAA are slightly higher in white matter relative to gray matter tissue [62]. Post-mortem studies have demonstrated that NAA is synthesized in neurons, transported into white matter and then catabolized into aspartate and acetate in oligodendrocytes via aspartoacylase [63]. NAA catabolism is therefore closely linked to myelin lipid metabolism, as it provides a very important source of acetate which is crucial for myelin lipid production and maintenance [64,65].

The assumption of abnormal myelin biosynthesis in schizophrenia, strongly supported by several different lines of evidence [8,9,66], can thus be examined by ^1^H-MRS studies as long as the targeted brain region involves a single tissue type (white matter) that allows a meaningful interpretation of findings in terms of the catabolic cycle of NAA.

Noteworthy for this study, the cannabinoid receptors CB1 are present on astrocytes and oligodendrocytes and may thus be implicated in the detrimental impact of early adolescent cannabis use by affecting the trajectory of white matter development in the critical period of adolescence [4,53].

##### ^1^H-MRS: technical limitation

Due to the low concentration (mM) of the neurochemicals detected by ^1^H-MRS, localized spectroscopy studies generally sample a relatively large brain volume and require averaging several acquisitions to build up a reasonable signal-to noise ratio from the brain volume of interest. As such, most previous ^1^H-MRS clinical studies have reported on NAA signals originating from both gray and white matter tissues taken together as a whole; unfortunately, this approach has prevented the interpretation of findings in regards to the specific anabolic and catabolic activities of NAA.

In this proposed study, we will sample a large brain volume comprised of 95% white matter. Our previous ^1^H-MRS data acquired in the same brain region has demonstrated that across more than 150 brain scans acquired with these anatomical landmarks, the mean (SD) fractional content of white matter was 95(2.8)% (unpublished). These ^1^H-MRS data will thus provide insight about the specific catabolic cycle of NAA in early phase schizophrenia and consequently, about the regional availability of acetate which is required for biosynthesis of myelin. In this context, regional levels of NAA can be considered a marker of myelin integrity.

##### Previous relevant ^1^H-MRS studies

In adolescent chronic cannabis users, reductions in NAA concentration levels were reported, relative to non-user controls, in the anterior cingulate region encompassing mainly gray matter [67]. Levels of NAA might also be altered in schizophrenia but findings are inconsistent across studies, thus inconclusive. Indeed, if we compile previous ^1^H-MRS studies of schizophrenia while selecting studies with the best contemporary methods (those that referenced neurochemical levels to internal water and that used a sample size of 20 subjects or more in each group in order to decrease probabilities of “noise discoveries”) [68,69], no consensus can be reached in the current literature [70].

In the frontal/prefrontal regions of the brain, the focus of this study, some ^1^H-MRS studies have reported that concentrations were reduced in established schizophrenia relative to healthy controls for levels of NAA [71-74], and age-adjusted NAA [75]. On the other hand, several other studies have reported normal levels of NAA in the same frontal/prefrontal regions in never treated first episode psychosis [76], medicated first episode psychosis [77,78], and established schizophrenia [79-86]. These studies, for the most part, sampled a brain volume of interest encompassing both gray matter and white matter tissue types, consequently precluding any specific interpretation of findings in terms of the precise anabolic or catabolic cycle of NAA. Obviously there is a need to search for confounding variables that might impact on the current mixed ^1^H-MRS findings reported across population samples and laboratories. Early adolescent onset of regular cannabis use certainly has the potential to be one such factor [3,5].

#### Transverse (T_2_) relaxation time constants

Another neuroimaging modality involved in this study targets the same white matter brain region, while maintaining the focus at the cellular level. The transverse relaxation time constants of regional tissue water (involving both intracellular and extracellular tissue water) and of NAA (intracellular) provide an index of the integrity of the microcellular environment of the brain region studied. In fact, T_2_ relaxation time constants are dependent on the morphological parameters of cell size and cell packing density in the brain region studied; they also reflect intracellular molecular mobility as they are dependent on the frequency of molecule-microenvironment interactions [87]. As such, prolonged T_2_ time constants are associated with reduced cell densities.

Transverse time constants of NAA in the context of ~95% white matter tissue will provide an index of intracellular density of cells in this brain region, oligodencrodytes being an important target among these cells [65]. Transverse time constants of water will provide an index of intracellular plus extracellular cell packing densities without differentiation of which type of cells are present in the specific region of interest.

##### Previous studies

Different from DTI and ^1^H-MRS studies of schizophrenia, the studies assessing T_2_ relaxation time constants in this illness have, to this date, reported consistent findings. The T_2_ time constants of water were found to be prolonged relative to healthy controls in prefrontal white matter [75,88] and anterior corpus callosum [89], in adults with established schizophrenia [75,88,89] and first episode psychosis [89]. No differences were observed between adults with first episode psychosis and those with established schizophrenia [89]. These findings altogether support the assumption of abnormal axonal milieu and myelin structures in schizophrenia.

Transverse relaxation time constants of NAA, on the other hand, were found to be shortened relative to healthy controls in prefrontal white matter of adults with established schizophrenia [75,88] and first episode psychosis [89], thus supporting the assumption of increased intracellular (oligodendrocytes) cell density. In gray matter (anterior cingulate cortex), T_2_ time constants of NAA were also shortened in adults with schizophrenia relative to healthy controls but the group difference did not reach statistical significance [90].

Because of the consistency of reported findings with these particularly sensitive neuroimaging indices of cell packing densities, T_2_ relaxation time constants acquired in this proposed study will be used as an anchor point against which DTI and ^1^H-MRS measures will be interpreted. The aim is to generate, from these in vivo data, a plausible interpretation of the specific cellular abnormalities associated with schizophrenia, which might differ from those associated with early adolescent onset of regular cannabis use.

Noteworthy for this project, the T_2_ relaxation time constants of NAA and tissue water have never been used to help differentiate the detrimental impact of cannabis use from the white matter cellular abnormalities associated with schizophrenia. We expect that these sensitive measures of intracellular and extracellular cell packing densities will be related to DTI (FA) values, as water diffusivity is also influenced by cell densities [24].

### Aims

We propose a multimodal neuroimaging study of frontal white matter microstructure in patients in the early phase of schizophrenia, while taking into account the detrimental impact of early adolescent onset of regular cannabis use. The brain region of interest is the left SLF from which FA values, NAA levels as well as T_2_ relaxation time constants of tissue water and of NAA will be measured, providing novel insight into the specific cellular pathology associated with early phase schizophrenia, and into the potential confounding impact of early adolescent onset of regular cannabis use.

## Methods/Design

### Sample size and groups/subgroups

This study will involve 240 participants overall. The two main conditions are a) young adults in their early phase of schizophrenia (n = 120) and b) healthy controls (n = 120). Each condition will be further subdivided into three subgroups (n = 40 each) based on age at initiation of regular cannabis use: a) prior to age 17, b) after age 17, or c) with no lifetime exposure or very minimal experimentation with cannabis.

### A priori premises

1. How are we going to interpret the findings?

The particular neuroimaging indices that will show anomalies relative to normative data (non-user healthy controls) in regards to each of the four potential situations outlined below will permit a meaningful micro-structural interpretation of the specific and potentially different cellular abnormalities existing in each group and subgroup of participants.

a) Given the specific online selection of white matter tissue, reduction in regional levels of NAA will lead to the assumption of insufficient availability of acetate, which is the main ‘building block’ necessary for myelin repair and maintenance. Thus, we would assume reduced integrity of *myelin sheaths*.

b) In the case of reduction in FA values, we would assume reduced integrity of *axonal fibres* in this same brain region (more disorganized axonal fibres). Integrity of myelin sheaths has less impact on FA values than integrity of axonal fibres.

c) In the case of prolonged T_2_ relaxation time constants of regional tissue water, we would assume reductions in intracellular and extracellular cell packing density of *axonal fibres* (myelin water is not included in this measure; see next section).

d) T_2_ time constants of NAA are sensitive to intracellular density; as such, shorter T_2_ time constants would yield the assumption of greater intracellular density of regional white matter cells, *oligodendrocytes* being involved.

2. Specific hypotheses:

a) In terms of T_2_ relaxation time constants of NAA and of regional tissue water:

•In the clinical group (n = 120), T_2_ time constants of NAA will be reduced relative to healthy controls (n = 120), while T_2_ time constants of tissue water will be prolonged (replication data) [75,88,89]. In addition, the clinical subgroup of early cannabis users (n = 40) will display a greater level of deviation from normative data (non-user healthy controls; n = 40) compared with the two other clinical subgroups (novel data).

•In the healthy control group, similar findings (as above) will be observed in early cannabis users (n = 40) relative to non-users (n = 40), but not in late cannabis users (n = 40) relative to non-users (novel data).

b) In terms of FA values and NAA levels: We expect these two measures to correlate with each other [82].

•In the clinical group (n = 120), reduced FA values and NAA levels might or might not be observed relative to healthy controls (n = 120), as previous findings are mixed and inconclusive; however, the clinical subgroup of early cannabis users will display reduced FA values and NAA levels relative to normative data and relative to the two other clinical subgroups (novel data).

•In healthy controls, reduced FA values and NAA levels will be found in early cannabis users relative to non-users (replication data) [51,53-56], but not in late cannabis users relative to non-users (novel data).

3. Associations between neuroimaging indices:

We expect that correlations between abnormalities in neuroimaging indices will be stronger in early cannabis users with schizophrenia relative to non-users with schizophrenia and relative to early cannabis users without symptoms of schizophrenia.

4. Associations with symptom/function measures:

We expect that in the clinical group, abnormalities in neuroimaging indices will correlate with more pronounced clinical/functional abnormalities according to symptom/function measures (see section Questionnaires and interviews, below). There are few studies investigating the relationship between different aspects of white matter cellular integrity and symptom/function measures in early phase schizophrenia. These analyses will be exploratory and will be used for the generation of hypotheses for future studies.

### Statistical power analyses

To our knowledge, this study is the first one to compare and contrast four cellular neuroimaging indices acquired from the exact same brain region, while targeting a single tissue type. As such, these data will help establish statistical power calculations for future studies. We computed power estimations using data from studies with contemporary neuroimaging methods and relatively good sample sizes, while selecting those studies that were very close to our own research question. Our purpose was to ensure that our planned sample size was reasonable even in this context of a pioneer study.

From previous data reporting T_2_ relaxation time constants of NAA in patients with schizophrenia relative to healthy controls [75], we estimated that a sample size of 35 participants in each group would provide adequate power to detect differences between independent groups, with a two-tailed test at an alpha level of .05 and power of .8.

From previous data reporting T_2_ relaxation time constants of water in patients with schizophrenia relative to healthy controls [88], we estimated that a sample size of 23 participants in each group will provide adequate power to detect differences between independent groups.

From frontal DTI FA values previously reported in patients with schizophrenia who started cannabis use prior to age 17 versus healthy non-users controls [60], sample size calculations revealed that 23 participants in each group will yield adequate power to detect group differences.

With the reduced levels of NAA in anterior cingulate previously reported in adolescent marijuana users versus non-user controls [67], computations yielded a sample size of 24 participants in each group in order to have adequate power.

Our planned sample size of 40 participants per subgroup will thus yield adequate power for all planned analyses.

### Operational definitions

•Early phase schizophrenia: less than 5 years since diagnosis of psychosis with initiation of appropriate medical treatment [20].

•Regular cannabis use: usage occurring on 3 or more days per week, maintained for a period of 6 months or more [34].

•Early adolescent onset of cannabis use: start of regular cannabis use prior to age 17 years old [33,60].

•Minimal or non-cannabis users: people who are cannabis-naive or who had minimal experimentation with cannabis (less than 10 experimentations over lifetime) [57].

### Recruitment and diagnosis

Recruitment of patients will be conducted at the Nova Scotia Early Psychosis Program (NSEPP). Currently NSEPP has about 220 individuals who are active in the clinic and within 5 years of illness onset (meeting our criteria); approximately 60-70% of these patients have a history of cannabis use, as assessed at time of referral. Approximately 50 new incoming patients are accepted at the clinic every year, adding to the current pool of patients. Recruitment of healthy controls will be conducted through advertisements. Diagnosis of patients (using DSM-IV) will be confirmed by consensus between the treating psychiatrist and one of the authors (PT).

### Inclusion and exclusion criteria

Healthy controls will be 19–35 years of age; with no lifetime diagnosis of psychiatric disorder; healthy; and taking no prescribed medications. They will have no first degree relatives (sibling, mother, or father) with a lifetime diagnosis of psychosis or bipolar disorder. They will be matched with patients in regards to age, gender and history of cannabis use. Patients will be 19–35 years of age and within five years of diagnosis of schizophrenia. They will be taking appropriate antipsychotic medications (these will be recorded and tested as potential confounding variables). Participants will be excluded if they have a lifetime history of a) more than minimal experimentation with illicit drugs other than cannabis (e.g., cocaine, ecstasy) or b) more than low risk alcohol consumption as behaviourally defined by the Canada’s Low-Risk Alcohol Drinking Guidelines [90]; we estimated that approximately 10% of patients in our clinic will be excluded based on this lifetime history.

### Ethics approval and financial support

Full ethics approval to conduct this study was received from the Capital Heath Research Ethics Board. Capital Health is a public provider of health care services in Halifax, Nova Scotia, Canada. Ethics approval was also obtained from the IWK Research Ethics Board, as the scanner is located in this hospital. The IWK Health Centre is a public provider of health care in Halifax. Each participant will be fully informed of the study prior to signing the consent form. Seed funding for this study was obtained from the Department of Psychiatry Research Fund at Dalhousie University, Halifax. This study is also financially supported by the Dr. Paul Janssen Chair in Psychotic Disorders (P. Tibbo).

### Questionnaires and interviews

•The Structured Clinical Interview for the Diagnostic and Statistical Manual of Mental Disorders Axis 1 (SCID-1) [91] is a semi-structured interview used for diagnosis.

•The Structured Clinical Interview for the Positive and Negative Syndrome Scale (SCI-PANSS) [92] assesses 30 different symptoms associated with schizophrenia [93], grouped into three subscales: The Positive, Negative and General Symptoms subscales.

•The Personal and Social Performance scale (PSP) was developed to measure social functioning in schizophrenia, separate from psychological symptoms [94].

•Severity of anxiety and mood symptoms is assessed with the Beck Anxiety Inventory (BAI) [95] and the Calgary Depression Rating Scale for Schizophrenia [96].

•Detailed information about past and current use of all types of illicit drugs, alcohol, and cigarette smoking is collected using a custom Drug Questionnaire, which includes all the questions provided in the SCID but organized in a much more detailed way. Cumulative usage of cannabis will be estimated (in grams) for two time periods: before age 17 (when applicable) and cumulative lifetime.

### Neuroimaging

#### MR online acquisitions

**Figure 1:**
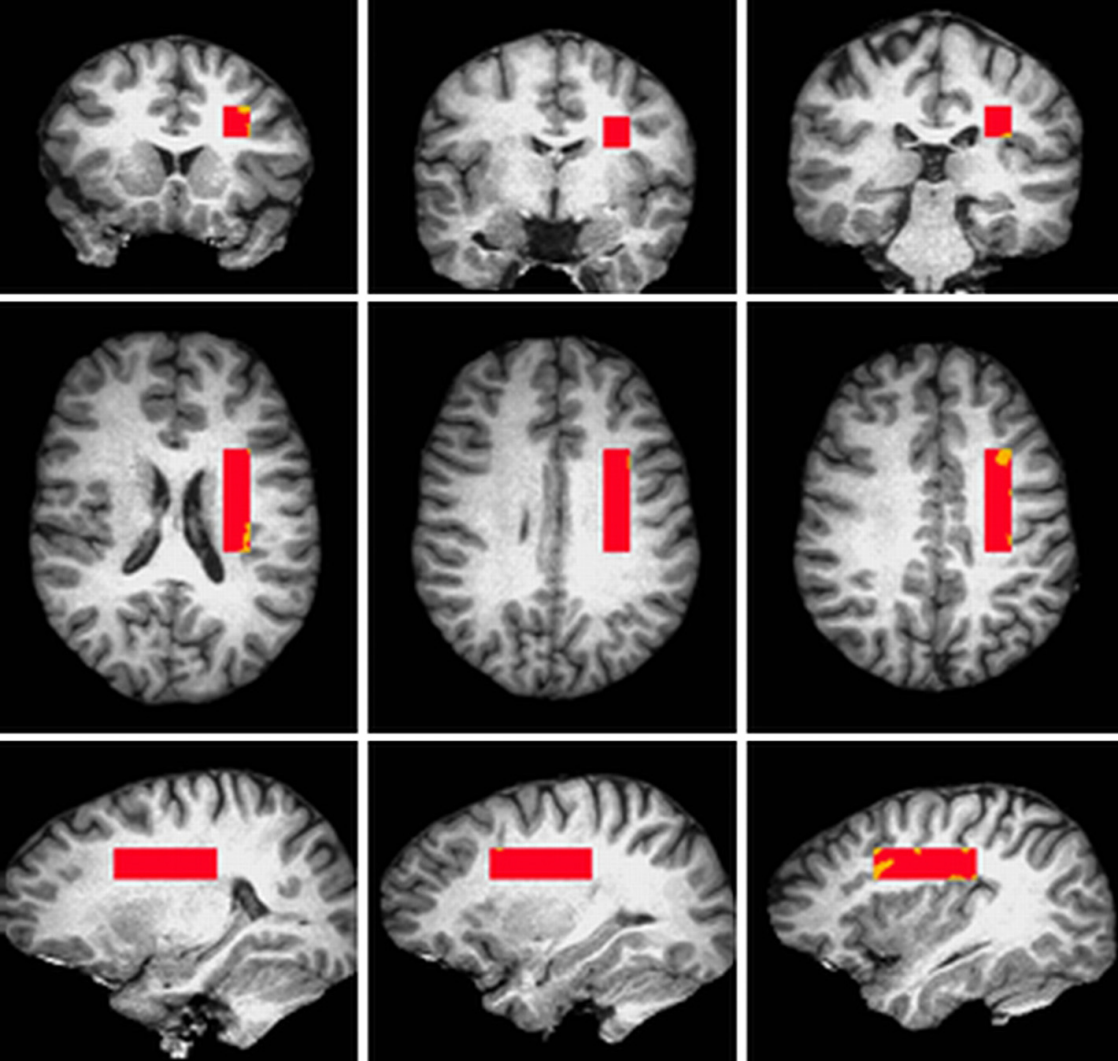
**Online placement of the **^**1**^**H-MRS volume of interest.** Note. The volume is parallel to the AC-PC line. Its posterior border is aligned 3 mm posterior to the point of posterior commissure using the sagittal view. Its inferior border is placed axially on the first slice where the corpus callosum meets from the two hemispheres. In the right/left direction, it is centered on the white matter fibre tract using the inferior axial slice.

Neuroimaging data will be acquired with a GE 1.5 Tesla MRI and a multi-channel head coil. MR acquisition parameters are outlined in Table 1. A 6.5 cm volume of interest (VOI) is prescribed in the left dorsal frontal white matter, immediately anterior to the rostral part of the inferior parietal lobe. VOI dimensions are 45 mm (A/P) by 13 mm (S/I) and 11 mm (R/L) (Figure 1). Relaxometry acquisitions for NAA start at a TE of 80 ms, in order to minimize the contribution from macromolecules into the estimation of neurochemicals (which would present at shorter TE times). Relaxometry acquisitions for the water files start at a TE of 50 ms, in order to minimize the contribution from the myelin water signal into the estimations of tissue water relaxation time**s**[87].

**Table 1:** Neuroimaging acquisition sequences

**Type**	**Parameters**	**Min**
Localizer and calibration		2
3D SPGR T_1_-weighted, for online placement of VOI and its offline tissue segmentation	256 x 256 matrix; 170 sagittal slices; 1 mm isotropic resolution, no inter-slice gap; TR = 11.3 s; TE = 4.2 ms; flip angle = 20 deg.	7
^1^H-MRS volume of interest (VOI)	Online VOI placement; shimming (values are carried over to each subsequent ^1^H-MRS acquisition)	6
NAA concentration levels and T_2_ time constants of NAA	PRESS; TR = 3 s; TEs = 80, 120, 180, 350, 600 ms; NEX = 64	25
T_2_ time constants of water	TR = 10 s; TEs = 50, 60, 80, 120, 180, 350, 600, 800, 1000 ms; NEX = 4	20
Diffusion-weighted images	TR = 8.5 s; TE 80–90 ms; flip angle = 90 deg; 54 non-collinear diffusion weighting directions, b-factor of 1000 s/mm^2^; 6 acquisitions, b-factor of ~ 0 s/mm^2^; 256 x 256 matrix; 260 FOV; 1.02 x 1.02 x 3 mm^3^ voxels; NEX = 1; acquisition of field maps.	25

#### MR offline analyses

##### ^1^H-MRS data

Offline analysis of the neurochemical spectra are performed with the program fitMAN [97]. The following signals will be quantified: *N*-acetylaspartate plus *N*-acetylaspartylglutamate (NAA), choline-containing compounds (Cho), and creatine plus phosphocreatine (tCr). We will compile all the data acquired, even when not part of the main research question. At 1.5 Tesla however, it is impossible to biologically meaningfully interpret findings from the Cho signal, as its anabolic component (phosphocholine; PC) cannot be resolved separately from its catabolic component (glycerophosphocholine; GPC).

Neurochemical concentration levels will be adjusted to account for the fractional content of tissue types within each VOI, according to their known variation in this respect [62], and they will be referenced to the estimation of internal water signal extrapolated to TE = 0 ms. For each individual participant, neurochemical levels will be corrected for T_2_ induced signal losses.

Noteworthy, the fitMAN program can be used to analyse spectra in the time domain, therefore allowing the elimination of the tail of the free induction decay curve (where mainly noise remains) from the fitting, which strategy strikingly increases the signal-to-noise ratio (SNR) of the spectra in the frequency domain. The model function used is Equation 1 in Bartha et al. (1999) [97], with parameters for both zero- and first-order phase. As a result, there is no need to perform zero- or first order-phasing of the data prior to quantification in the time domain; these parameters are estimated as part of the fitting process. When fitting in the time domain, we simply specify the time interval over which to perform the minimization; therefore, eliminating data points at the beginning or end of the FID becomes straightforward. For spectral fitting, we include data points that range from 1 to 512.

**Figure 2:**
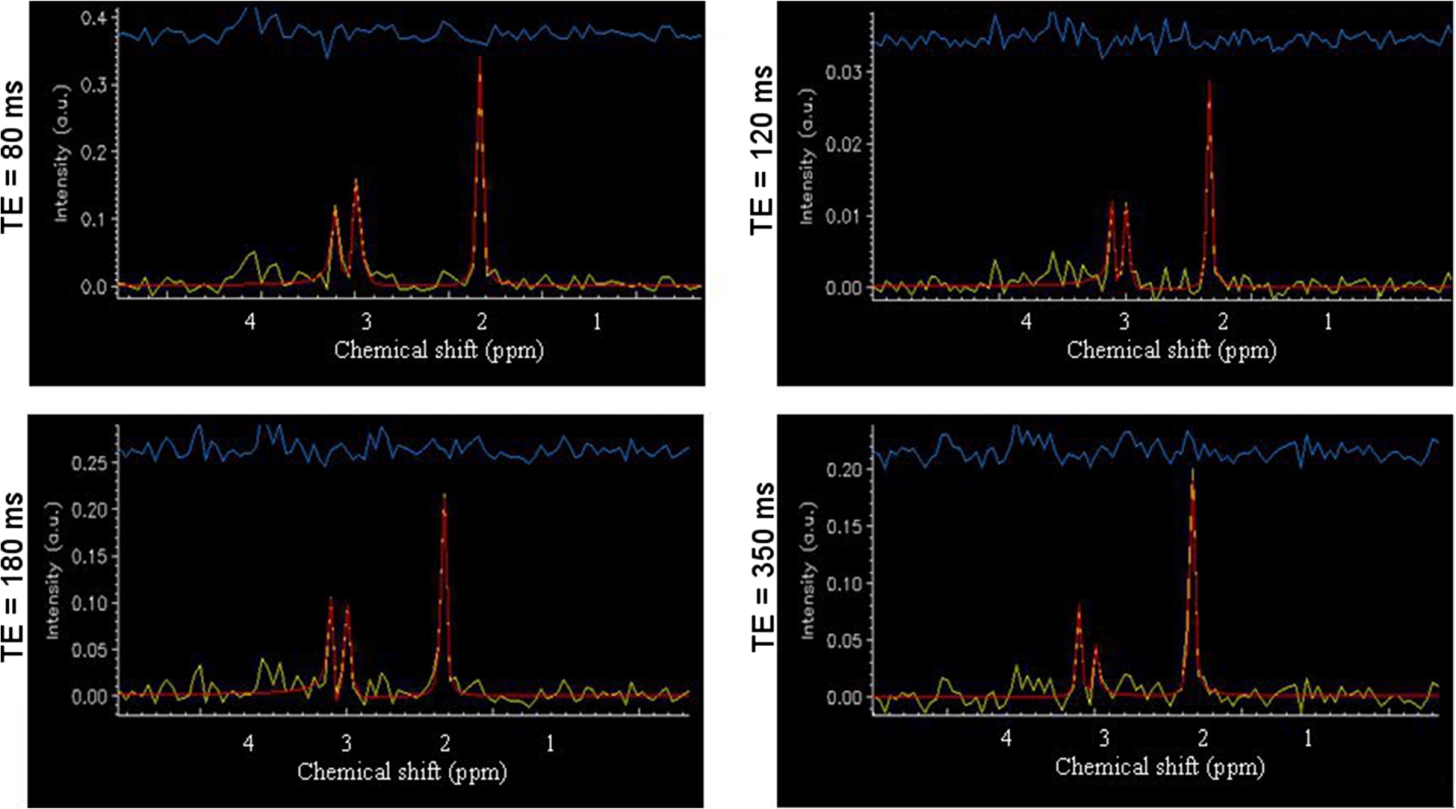
**Spectral profiles acquired at different times of echo (TE).** Note. Yellow: raw data; blue: residuals; red: fitted data. No data filtering has been applied. In the time domain, the number of data points included in the fits ranged from 1 to 512.

Prior knowledge basis set spectra are not required as the three main singlets of the spectral profiles are quite robust. The quality criteria for ^1^H-MRS data to be retained for statistical analyses are the following: signal-to-noise ratio (SNR) of 15 or greater, computed from the amplitude of NAA divided by the standard deviation (SD) of the noise; linewidth (FWHM) of 8 Hz or less; and uncertainty in the estimation of the fit (Cramer Rao bounds) smaller than 10 %SD. Our preliminary data shows that SNR for NAA typically ranges from 20 to 55, depending on the specific TE used for acquisition; FWHM is 6 Hz or less; and % SD is consistently ≤ 5 (Figure 2).

##### T_2_ measurements

**Figure 3:**
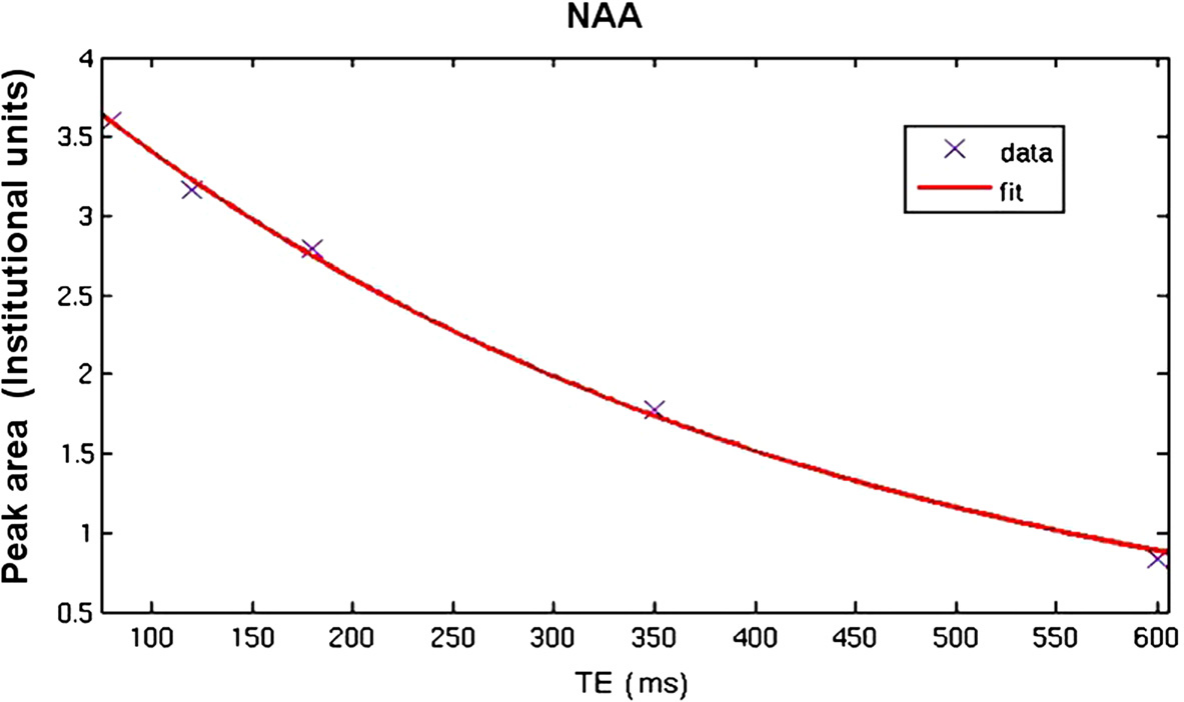
**NAA signal decay curve.** Note. NAA signal areas are fitted to a single exponential decay model using MATLAB.

**Figure 4:**
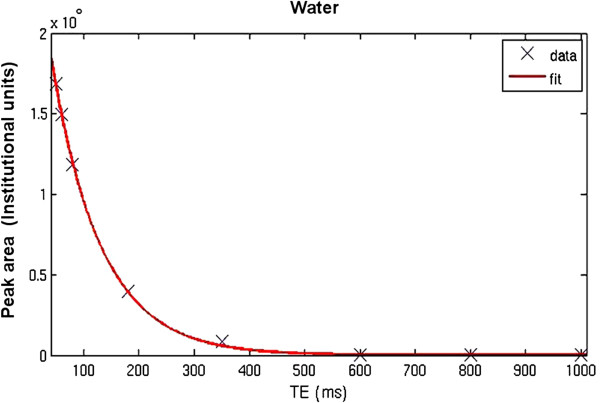
**Water signal decay curve.** Note. Water signal areas are fitted to a two-component decay model using MATLAB.

Area under the water signal is calculated from the frequency domain at each TE, using a MATLAB script ([98]. From the decrease in water signal amplitude as a function of TE, the corresponding T_2_ relaxation time constants are estimated as a curve fit of a two-component exponential decay using the Curve Fitting Tool in MATLAB. The decay curve of water could potentially involve three components: CSF (long T_2_), tissue water (intermediate T_2_), and myelin water (very short T_2_) [87]. With this study design however, signals from myelin water mostly decayed at the echo times sampled; the very short myelin component is therefore not identified by the fitting algorithm. As for NAA levels, signal loss (as a function of TE) has a better fit with a mono-exponential decay curve [99] (Figures 3 and 4).

#### DTI data

The scanner 3D coordinates used at time of ^1^H-MRS online acquisition will be used to precisely define a DTI region of interest encompassing the exact same brain volume that was prescribed for estimation of ^1^H-MRS neurochemical levels and T_2_ relaxation time constants. This strategy will permit the offline estimation of all neuroimaging indices (DTI, ^1^H-MRS and T_2_ time constants) from the exact same brain region, as precisely defined by online coordinates of ^1^H-MRS VOI placement.

Fractional anisotropy (FA) images are calculated using Bayesian estimation of diffusion parameters at each voxel. Images are spatially normalized using nonlinear registration to the MNI152 brain template. A mean FA image is created and thinned to generate a mean FA skeleton representing the maximum FA values of all tracts common to all participants. A threshold of 0.2 is applied to the skeleton to control for cross-subject variability.

The VOI described above is used to mask the FA skeleton image; tracts passing through this VOI are statistically analysed using nonparametric tract-based spatial statistics [100] using 10000 permutations. Threshold-free cluster enhancement [101] is used to correct for multiple comparisons. Statistical significance is determined at p < .05, corrected. Tracts are identified using the MRI Atlas of Human White Matter and the JHU DTI-based white matter atlases included with the FSL software: the ICBM-DTI-81 white matter labels atlas and the JHU white matter tractography atlas [102,103].

### Statistical analyses

All statistical analyses will be two-tailed and computed with SPSS version 17; alpha will be set at p < .05 unless otherwise specified.

Testing hypotheses 1 and 2:

A multivariate analysis of variance (MANOVA) will be computed. Two between-group factors will be entered in the model: Group with two levels (clinical and controls), and Subgroup with three levels (Early, Late, and Minimal cannabis users). The four dependent variables will be T_2_ time constants of water and of NAA, FA values, and NAA levels. Analyses of variance (ANOVAs) and then t tests will be used to follow-up on significant main effects and interaction effects.

Testing hypothesis 3:

Potential significant interaction effects between the two factors entered in the MANOVA will be used as a basis to determine whether or not hypothesis 3 is supported. Further follow-up analyses of these significant interactions will involve Pearson’s correlations with relevant neuroimaging indices.

Testing hypothesis 4:

Exploratory analyses will involve Pearson’s correlations between neuroimaging variables and clinical variables; e.g., cumulative lifetime use of cannabis, gender, current age, age at onset of psychosis, duration of untreated psychosis, current stage of illness, severity of symptoms, medication type, and length of time taking antipsychotic medications. A p value of .01 or smaller will be necessary for an association to be considered for discussion.

## Discussion

We hereby propose to differentiate the detrimental impact of early adolescent cannabis use from the cellular changes associated with schizophrenia, in order to refine the current understanding of the specific cellular mechanisms involved in white matter abnormalities in the early phase of schizophrenia [64]. This comparison will also highlight the protective factors by which resiliency to cannabis use occurs; that is, not all cannabis users develop psychosis.

## Abbreviations

Cho: Choline-containing compounds; MD: Mean diffusivity; DTI: Diffusion tensor imaging; NAA: N-acetylaspartate; FA: Fractional anisotropy; SLF: Superior longitudinal fasciculus; FWHM: Full width at half maximum of peak; tCr: Creatine plus phosphocreatine; 1H-MRS: Proton magnetic resonance spectroscopy; TE: Time of echo; MANOVA: Multivariate analysis of variance; TR: Time of repetition.

## Competing interests

The authors declare that they have no competing interests.

## Authors’ contributions

DB made a substantial contribution to the conception and design of the proposal. DB and JC made a substantial contribution to the initial writing of the draft proposal. JC is recruiting and screening participants. JC and DB are acquiring data and performing MR offline analyses. DM made a substantial contribution to all MR technical aspects of this proposal namely, pre-testing of MR data quality; computing of the decay curves and T_2_ relaxation time constants; and all aspects of compilation of MR data. RB provided his program for ^1^H-MRS offline analyses (fitMAN) and also provided technical support for the program in the context of 3-peak fits at 1.5T, along with extensive teaching about offline analyses. CH has designed the relaxometry parameters for acquisition and offline analyses; he also provided a custom-made program to assess the decay curves. RB and CH made a substantial intellectual contribution to the writing of the ^1^H-MRS technical part of the proposal. AN has designed the DTI section of the proposal in terms of data acquisition parameters and offline analyses as well as writing of this technical section. SS brought expertise about the addiction part of this study. PT provided help with recruitment and diagnosis of patients, and he supported the research as the Director of the NSEPP and as the Dr. Paul Janssen Chair in Psychotic Disorders. All authors have made intellectual contributions to the writing and editing of the full study proposal. All authors read and approved the final manuscript.

## Pre-publication history

The pre-publication history for this paper can be accessed here:

http://www.biomedcentral.com/1471-244X/13/264/prepub
